# Accuracy of Autonomous Artificial Intelligence-Based Diabetic Retinopathy Screening in Real-Life Clinical Practice

**DOI:** 10.3390/jcm13164776

**Published:** 2024-08-14

**Authors:** Eleonora Riotto, Stefan Gasser, Jelena Potic, Mohamed Sherif, Theodor Stappler, Reinier Schlingemann, Thomas Wolfensberger, Lazaros Konstantinidis

**Affiliations:** Hôpital Jules Gonin, 1004 Lausanne, Switzerland; stefan.gasser@fa2.ch (S.G.);

**Keywords:** diabetic retinopathy, artificial intelligence, diabetes, IDX-DR

## Abstract

**Background:** In diabetic retinopathy, early detection and intervention are crucial in preventing vision loss and improving patient outcomes. In the era of artificial intelligence (AI) and machine learning, new promising diagnostic tools have emerged. The IDX-DR machine (Digital Diagnostics, Coralville, IA, USA) represents a diagnostic tool that combines advanced imaging techniques, AI algorithms, and deep learning methodologies to identify and classify diabetic retinopathy. **Methods:** All patients that participated in our AI-based DR screening were considered for this study. For this study, all retinal images were additionally reviewed retrospectively by two experienced retinal specialists. Sensitivity, specificity, positive predictive value (PPV), negative predictive value (NPV), and accuracy were calculated for the IDX-DR machine compared to the graders’ responses. **Results:** We included a total of 2282 images from 1141 patients who were screened between January 2021 and January 2023 at the Jules Gonin Eye Hospital in Lausanne, Switzerland. Sensitivity was calculated to be 100% for ‘no DR’, ‘mild DR’, and ‘moderate DR’. Specificity for no DR’, ‘mild DR’, ‘moderate DR’, and ‘severe DR’ was calculated to be, respectively, 78.4%, 81.2%, 93.4%, and 97.6%. PPV was calculated to be, respectively, 36.7%, 24.6%, 1.4%, and 0%. NPV was calculated to be 100% for each category. Accuracy was calculated to be higher than 80% for ‘no DR’, ‘mild DR’, and ‘moderate DR’. **Conclusions:** In this study, based in Jules Gonin Eye Hospital in Lausanne, we compared the autonomous diagnostic AI system of the IDX-DR machine detecting diabetic retinopathy to human gradings established by two experienced retinal specialists. Our results showed that the ID-x DR machine constantly overestimates the DR stages, thus permitting the clinicians to fully trust negative results delivered by the screening software. Nevertheless, all fundus images classified as ‘mild DR’ or greater should always be controlled by a specialist in order to assert whether the predicted stage is truly present.

## 1. Introduction

Diabetic retinopathy (DR) is a leading cause of visual impairment and blindness among individuals with diabetes mellitus [1]. This condition arises due to prolonged high blood sugar levels, causing damage to the blood vessels in the retina. Early detection and timely intervention are crucial in preventing significant vision loss and improving patient outcomes [2]. This highlights the necessity for advancements in medical diagnostics.

The emergence of artificial intelligence (AI) and machine learning technologies has revolutionized numerous fields, including healthcare. One notable development in this area is the IDX-DR machine, developed by Digital Diagnostics in Coralville, IA, USA. This machine marks a significant milestone, as it became the first FDA-authorized autonomous AI diagnostic system in any medical field. The FDA approved the use of this system by healthcare providers to detect more than mild diabetic retinopathy and diabetic macular edema, underscoring its potential in transforming diabetic eye care [2].

The IDX-DR machine represents a cutting-edge diagnostic tool that combines advanced imaging techniques, AI algorithms, and deep learning methodologies to identify and classify diabetic retinopathy [3,4,5]. This innovative system leverages high-resolution retinal imaging and sophisticated computational analysis to offer a non-invasive, rapid, and reliable solution for early DR detection. This assists healthcare professionals in making timely and informed clinical decisions. One of the fundamental components of the IDX-DR machine is its advanced imaging system. Employing state-of-the-art techniques, such as optical coherence tomography (OCT) and fundus photography, the machine captures detailed images of the retina. These images enable a comprehensive assessment of retinal structures, including blood vessels, microaneurysms, exudates, and other pathological features associated with DR [6]. The high-resolution images generated by the IDX-DR machine serve as the foundation for subsequent AI-driven analysis, ensuring accuracy and reliability in diagnosing various stages of DR.

The AI outputs classify the images based on the International Clinical Disease (ICDR) Severity Scale [7]. This scale is instrumental in identifying the severity of diabetic retinopathy. According to the ICDR scale, mild DR is characterized by the presence of only microaneurysms. Moderate DR is identified if more than just microaneurysms, including hemorrhages, can be observed. Severe DR is indicated by the presence of extensive intraretinal hemorrhages in four quadrants, venous beading in more than two quadrants, prominent intraretinal microvascular anomalies (IRMA) in one or more quadrants, and no signs of proliferative DR [7]. This classification system ensures a standardized approach to diagnosing and managing diabetic retinopathy, facilitating effective communication and treatment planning among healthcare providers.

Numerous studies have focused on evaluating the sensibility and sensitivity of IDx-DR. Van der Heijden AA et al. reported a sensitivity of 68% and a specificity of 86% in a 1410-patient screening [8]. In another study by Abramoff et al., the machine demonstrated a sensitivity of 87.2% and a specificity of 90.7% in detecting more than mild disease among 900 patients with diabetes [2]. Shah et al. observed a sensitivity of 100% and a specificity of 82% in detecting referable DR in a population of 2680 subjects [9]. Additionally, Ting et al. reported a sensitivity of 90.5% and a specificity of 91.6% in detecting referable diabetic retinopathy, and a sensitivity of 100% and a specificity of 91.1% in detecting vision-threatening diabetic retinopathy [10]. Lastly, Peris-Martínez C. et al. found a sensitivity of 100% and a specificity of 81.82% in detecting derivable DR, and a sensitivity of 100% and a specificity of 94.64% in detecting diabetic retinopathy threatening to vision [11]. These studies collectively underscore the efficacy and reliability of the IDX-DR machine in identifying various stages of diabetic retinopathy with high accuracy.

In our study, we aim to determine the performance of the IDX-DR machine in correctly identifying different degrees of diabetic retinopathy. We specifically focus on evaluating the device’s efficiency across various stages of DR. By doing so, we hope to provide comprehensive insights into the capabilities and limitations of this AI-driven diagnostic tool. Our research is intended to contribute to the growing body of evidence supporting the use of AI in medical diagnostics, particularly in the early detection and management of diabetic retinopathy. This has the potential to significantly improve patient outcomes and reduce the burden of diabetic eye disease on individuals and healthcare systems alike.

## 2. Methods

In this study, we gathered images from the clinical records of patients participating in an AI-based diabetic retinopathy (DR) screening program at the Jules Gonin Eye Hospital in Lausanne, Switzerland. Such screening uses a full-size fundus camera and an IDX-DR machine (Digital Diagnostics, Coralville, IA, USA) to detect early or advanced signs of DR in diabetic patients.

Both eyes of a patient are evaluated by the IDX-DR machine to provide a final unique classification based on the International Clinical Disease (ICDR) Severity Scale. The IDX-DR machine is set up to provide the final grade based on the eye with the most advanced signs of DR.

An AI-based DR screening program was initiated in 2021 at the Jules Gonin Eye Hospital in Lausanne, Switzerland. All new diabetic patients referred to our dedicated diabetic retinopathy clinic undergo a thorough clinical examination. This examination includes wide-field retinal photographs and optical coherence tomography (OCT) imaging, conducted by an experienced team of retinal specialists, highly trained certified optometrists, and retinal imaging technicians. Patients were not excluded based on age, sex, or general illnesses; however, individuals with known previous retinal pathologies and those with corneal or lens defects potentially compromising image quality were managed through regular consultations rather than being included in the AI-based screening program.

All patients for whom no diabetic retinopathy is detected and who additionally have equilibrated systemic risk factors, and who are not considered at high risk of developing diabetic retinopathy, participate in our AI-based DR screening program at the two following consecutive follow-up appointments. Patients presenting signs of DR are referred to retinal specialists to ensure they receive specialized care tailored to their specific needs, including timely interventions and ongoing monitoring as recommended by clinical guidelines.

At all appointments, retinal imaging, including OCTs and retinal photographs, are evaluated and graded additionally and independently by two experienced retinal specialists. Angiography is performed in doubtful cases to elucidate the presence or absence of DR. Discrepancies between physician interpretations are meticulously discussed among the team of professionals involved in the study to reach a consensus.

For this study, all retinal images were additionally reviewed retrospectively.

All patients that participated in our AI-based DR screening were considered for this study.

Images that were classified as being of insufficient quality by the IDX-DR machine were excluded from this study.

For each IDx-DR degree classification, outcomes were defined as true positives if the grade identified by the machine was the same of that identified by the clinicians. Images that were correctly classified by the machine as not having a certain degree of DR, or as having an inferior DR grade to the one tested, were defined as true negatives. Images that were classified by the machine as having a certain DR degree, but that were in fact classified as being of a lower grade by the clinicians, were defined as false positives. Conversely, images that were classified by the machine as having a certain DR degree, but that were in fact classified as being of a higher grade by the clinicians, were defined as false negatives.

Based on the ICDR classification, sensitivity, specificity, positive predictive value (PPV), negative predictive value, and accuracy were calculated for the IDX-DR machine compared to the graders’ responses. The above-mentioned proportions were calculated for each categorization (no DR, mild DR, moderate, and severe DR). The analyses were conducted in R.

## 3. Results

The IDx-DR machine analyzed a total of 2700 images from 1350 patients who were screened between January 2021 and January 2023. This screening period allowed for a comprehensive assessment of the AI-based system’s performance over a significant timeframe, providing ample data for real-life statistical analysis.

Among them, 418 images from 209 patients were classified as ‘insufficient quality’ for analysis by the IDx-DR machine and were excluded from this study. These exclusions were necessary to ensure that the analysis was based on high-quality images, as poor-quality images could lead to inaccurate classifications and affect the overall reliability of the results.

After excluding the images deemed to be of insufficient quality, 2282 images from 1141 patients were finally included in this study.

Of these, 1590 images from 795 patients were classified as ‘absence of DR’ by the IDx-DR machine.

Notably, all these images were independently classified as ‘absence of DR’ by the two retinal specialists, demonstrating a high level of agreement between the AI system and human experts in identifying the absence of diabetic retinopathy.

The remaining 692 images from 346 patients were classified as mild, moderate, or severe diabetic retinopathy by the IDx-DR machine. Among these, 244 patients were classified as having ‘mild diabetic retinopathy’, 74 as having ‘moderate retinopathy’, and 28 as having ‘severe retinopathy’. However, the accuracy of these classifications varied when compared to the assessments made by the retinal specialists.

Among the 244 patients classified as having ‘mild DR’ by the IDx-DR machine, the physicians confirmed the diagnosis of ‘mild DR’ in only 60 patients. In the remaining 184 patients, the physicians detected no signs of diabetic retinopathy, highlighting a substantial number of false positives at this classification level.

**Figure 1:**
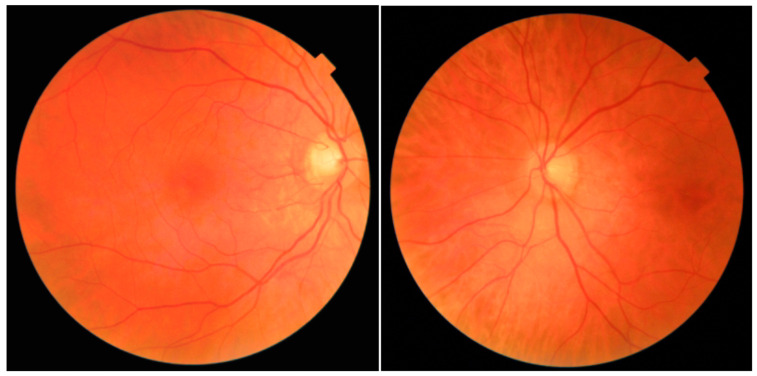
Image of bilateral fundus categorized as ‘Mild DR’ by screening software but showing no authentic signs of DR.

Figure 1 provides an illustrative example of a bilateral fundus categorized as ‘mild DR’ by the screening software, which, upon expert review, showed no authentic signs of DR. Among the 74 patients classified as ‘moderate DR’ by the machine, the physicians confirmed only one case of ‘moderate DR’. In contrast, they reclassified 42 patients as having ‘mild DR’ and 31 patients as having ‘no DR’. Finally, among the 28 patients classified as ‘severe DR’ by the IDx-DR machine, none were confirmed to actually have severe DR by the specialists. Instead, 4 patients were found to have no signs of DR, 21 were classified as having ‘mild DR’, and 3 were classified as having ‘moderate DR’. This significant discrepancy underscores the challenge of accurately identifying severe DR using the AI-based system.

A total of 219 patients with no signs of DR were wrongly classified as ‘mild DR’, ‘moderate DR’, or ‘severe DR’ by the IDx-DR machine. A total of 63 patients with signs of mild DR were wrongly classified as ‘moderate DR’ or ‘severe DR’. Three patients with signs of moderate DR were erroneously classified as ‘severe DR’. Thus, a total of 285 patients were found to have an absence of DR or a lower grade of DR compared to the proposed IDx-DR machine’s result. Among these 285 patients, 266 (93.3%) underwent a fluorescein angiography to confirm the DR classifications proposed by the physicians, adding an extra layer of validation to the human assessments.

The sensitivity and specificity of the IDx-DR machine in detecting ‘no DR’ were calculated to be, respectively, 100% and 78.4%. This indicates that while the machine is very good at identifying the absence of DR when it truly is not present (sensitivity), it has a somewhat lower ability to correctly identify all true negatives (specificity).

The Positive Predictive Value (PPV) was measured to be 36.7%, indicating that only a little over one-third of the patients classified as having no DR by the machine were correctly identified. The Negative Predictive Value was measured to be 100%, showing that when the machine identifies a patient as not having DR, it is almost always correct. The accuracy was calculated to be 80.8%.

The sensitivity and specificity of the IDx-DR machine in detecting ‘mild DR’ were calculated to be 100% and 81.2%, respectively. The Positive Predictive Value (PPV) was measured to be 24.6%, suggesting that less than one-quarter of patients classified as having mild DR by the machine were correctly identified. The Negative Predictive Value was measured to be 100%. The accuracy was calculated to be 82.3%.

The sensitivity and specificity of the machine in identifying ‘moderate DR’ were calculated to be 100% and 93.4%, respectively. However, the Positive Predictive Value (PPV) was measured to be very low, at 1.4%, indicating that almost none of the patients classified as having moderate DR by the machine were correctly identified. The Negative Predictive Value was measured to be 100%. The accuracy was calculated to be 93.4%.

Finally, the specificity of the IDx-DR machine in detecting ‘severe DR’ was calculated to be 97.6%. Sensibility could not be calculated as no true positives were identified in this category. The Positive Predictive Value (PPV) was measured to be 0%, indicating no accurate severe DR diagnoses by the machine. The Negative Predictive Value was measured to be 100%. Accuracy could not be calculated as no sensibility was calculated.

The accuracy results are summarized in Table 1.

This detailed analysis highlights the strengths and limitations of the IDx-DR machine in detecting various stages of diabetic retinopathy. While the machine performs well in identifying the absence of DR, its ability to accurately classify mild, moderate, and severe DR requires further improvement.

## 4. Discussion

With the increasing prevalence of diabetic retinopathy (DR), the critical role of early detection and screening has become more evident than ever. Early intervention can significantly reduce the risk of severe vision loss and improve patient outcomes. To meet this urgent need, advanced technological solutions, particularly AI-driven software, have emerged as highly promising tools for DR screening. These AI systems are carefully designed and fine-tuned using a variety of extensive and diverse datasets to ensure accuracy and reliability.

The development of AI software for DR screening involves sophisticated algorithms that can analyze retinal images with remarkable precision, and identifying subtle signs of DR. These technologies can process large volumes of data rapidly, making them suitable for use in busy clinical settings where time and resources are often limited. Furthermore, AI systems offer the potential for standardizing DR screening, reducing variability in diagnosis, and ensuring that patients receive timely and accurate assessments.

However, before deploying such AI technologies in clinical practice, it is imperative to thoroughly evaluate their true potential and limitations. This involves rigorous testing and validation in real-world scenarios to confirm that the AI systems perform consistently and effectively across different populations. Understanding the strengths and weaknesses of AI-driven DR screening tools is crucial for their successful integration into healthcare systems.

The promise of AI in revolutionizing DR screening is substantial, but it requires careful consideration and validation to ensure that these technologies can deliver on their potential and contribute meaningfully to the early detection and management of diabetic retinopathy. By doing so, we can move closer to reducing the global burden of DR and improving the quality of life for millions of individuals affected by diabetes.

In this study, based in Jules Gonin Eye Hospital in Lausanne, we compared the autonomous diagnostic AI system of an IDX-DR machine detecting diabetic retinopathy to a human grading established by two experienced retinal specialists. Our aim was to evaluate the performance of the IDx-DR machine in identifying various stages of DR and to compare it to the gold standard of human assessment.

Sensitivity for no DR, mild DR, and moderate DR was calculated to be 100% in each category. However, sensitivity for severe DR could not be calculated due to the lack of data (true positives); there were no cases of severe DR confirmed by the specialists among the 1350 patients included in our study. As a matter of fact, no false negatives resulted from the IDx-DR screening among the 1350 patients, meaning that the machine did not miss any cases of DR that were later identified by the human graders. Our results correlate to those found by some previous studies. For instance, Shah et al. measured a 100% sensitivity and 82% specificity for detecting vision-threatening DR in a 2680-subject population [9]. Similarly, Ting et al. calculated a 100% sensitivity in detecting vision-threatening DR among 76,370 retinal images [10]. Finally, Peris-Martínez C et al. calculated a 100% sensitivity and a 81.82% specificity in detecting derivable DR and a 100% sensitivity and 94.64% specificity for diabetic retinopathy threatening to vision [11].

In our study, specificity varied between 78.4% and 97.6%, demonstrating a high capacity in classifying images as a negative or lower grade when the disease was not at the level of the tested category. This high specificity suggests that the machine is quite reliable in ruling out DR when it is not present.

A recent systematic review has analyzed a total of 35 studies and has confirmed that the IDx-DR machine demonstrates a high performance in detecting different stages of DR, with a pooled sensitivity of 93% to 97% and a pooled specificity of 90% to 98% [12]. Besides, concluding that the AI machine is likely on par with human clinicians, they also suggested that machine learning techniques have higher sensitivity than specificity in DR detection.

This pattern of higher sensitivity over specificity is advantageous in medical screening because it prioritizes false positives over false negatives, ensuring that fewer cases of DR are missed, which is crucial for preventing disease progression.

Such assertion is also sustained by our positive predictive value results. Besides being considerably low, varying between 36.7% and 0%, PPV decreased with increasing DR degree, proving once again that the ID-x DR machine tends to overestimate the DR stages. On the other hand, NPV was measured to be 100% in all four categories, showing that the IDx-DR can be fully trusted when delivering negative results.

With this study, we were able to evidence that the IDx-DR machine is an excellent tool to screen diabetic populations with unknown DR activity, as the AI-algorithm can fully be trusted when providing negative results. Nevertheless, all fundus images classified as a ‘mild DR’ or greater should always be controlled by a specialist in order to assert whether the predicted stage is truly present. The machine can therefore be safely utilized as a primary care tool to screen for potential signs of DR, thus constituting a cost- and time-efficient solution to select patients in real need of a full ophthalmic evaluation.

While clinical expertise is crucial for interpretation, collaborative efforts to refine AI algorithms through enhanced data diversity, clinician feedback, and advanced AI techniques could significantly contribute to reducing false positives and improving the reliability of AI in medical imaging. Advancements in AI techniques such as ensemble learning, where multiple models contribute to a final decision, or the integration of explainable AI frameworks, could also provide deeper insights into the AI decision-making process, further enhancing its utility in clinical settings.

Unfortunately, our study has some limitations. First of all, few patients with ‘moderate’ DR and no patients with ‘severe’ DR were present in our study, thus influencing the tested probability results in higher DR categories. Future research should investigate AI screening of these more advanced stages of DR to provide a more comprehensive evaluation of the IDx-DR machine’s performance across all severity levels. Including a broader spectrum of patients with varying stages of DR in subsequent studies could enhance our understanding of how AI screening performs across different disease severities. Nevertheless, while decreasing prevalence may affect PPV and NPV, sensitivity and specificity typically do not depend on the proportion of a condition within a population at a specific point in time. Moreover, we did not take into consideration patients that had images of insufficient quality, thus excluding part of the sample that would actually need to be reviewed by clinicians in a real-life situation.

In conclusion, IDx-DR software (version 2.3) for the detection of diabetic retinopathy (DR) is a reliable and effective screening method, and is particularly useful in primary care settings for excluding the presence of DR. The accuracy and consistency of this AI-driven tool make it an invaluable asset in the early identification of DR, ensuring that patients receive timely and appropriate interventions. One of the significant advantages of the IDx-DR software (version 2.3) is its high sensitivity, which helps in accurately identifying cases of DR and reducing the likelihood of missed diagnoses.

However, it is important to acknowledge that the current version of IDx-DR software (version 2.3) tends to favor false positives over false negatives. While this bias ensures a higher level of safety by minimizing the risk of missing a DR diagnosis, it can lead to an increased number of patients being referred for further evaluation, which may not always be necessary. Despite this tendency, the potential for the AI machine to improve its specificity in the future is promising. With advancements in AI algorithms and the incorporation of larger and more diverse datasets, the specificity of the IDx-DR software (version 2.3) can be enhanced, leading to more accurate screenings and reducing unnecessary referrals.

This improvement in specificity would allow clinicians to rely more confidently on software ratings, directly influencing their decisions regarding appropriate clinical management. As the technology evolves, the balance between sensitivity and specificity will become more refined, enabling the IDx-DR machine to provide even more precise assessments of DR. The continued refinement and validation of AI-based tools like the IDx-DR machine are essential for advancing their integration into clinical practice. Rigorous testing and real-world validation studies are necessary to ensure that these tools perform consistently across diverse populations and clinical environments.

The integration of AI-based screening tools into clinical practice offers numerous benefits beyond improved diagnostic accuracy. These tools can significantly enhance patient outcomes by facilitating early detection and intervention, ultimately reducing the progression of DR and its associated complications. Additionally, the use of AI-driven screening methods can optimize healthcare resources by streamlining the screening process and allowing healthcare providers to focus on patients who require immediate attention.

Moreover, the adoption of AI technologies in healthcare settings can contribute to a more standardized approach to DR screening. By minimizing the variability in diagnosis that can occur with human graders, AI tools like IDx-DR software (version 2.3) can ensure that patients receive consistent and accurate assessments, regardless of where they are screened. This consistency is crucial for managing chronic conditions like diabetes, where regular monitoring and timely intervention are key to preventing severe complications.

In summary, IDx-DR software (version 2.3) represents a significant advancement in the field of DR detection and screening. Its ability to accurately exclude the presence of DR in primary care settings makes it an invaluable tool for early intervention and improved patient outcomes. While the current tendency to favor false positives ensures safety, ongoing improvements in specificity will enhance the tool’s overall reliability. The continued refinement and validation of AI-based screening tools are vital for their successful integration into clinical practice, ultimately optimizing healthcare resources and standardizing the approach to DR screening across various settings.

## Figures and Tables

**Table 1:** A summary of the IDx-DR machine’s accuracy results.

	No DR	Mild DR	Moderate DR	Severe DR
Sensitivity	100%	100%	100%	-
Specificity	78.4%	81.2%	93.4%	97.6%
Positive Predictive Value	36.7%	24.6%	1.4%	0%
Negative Predictive Value	100%	100%	100%	100%
Accuracy	80.8%	82.3%	93.4%	-

## Data Availability

All data generated or analyzed during this study are included in this article. Further inquiries can be directed to the corresponding author.
